# FACTORS ASSOCIATED WITH HOSPITAL-TO-HOME DISCHARGE: USING A DECISION TREE ANALYSIS

**DOI:** 10.1093/geroni/igz038.3421

**Published:** 2019-11-08

**Authors:** Ji Yeon Lee, Kwang Joon Kim, Chang Oh Kim

**Affiliations:** Yonsei University, Seoul, Korea, Republic of

## Abstract

Because the number of older adults discharged home is increasing, a comprehensive geriatric assessment (CGA) becomes a helpful tool to make a decision about discharge destination. This study aimed to examine the predictors related with discharge destination using the component of CGA. We used decision tree analysis with the Classification and Regression Trees algorithm. Study participants were older adults over 65 years old who were admitted to geriatric department at a university-affiliated hospital in Korea. The older adults were assessed by multi-professional team at the initial stage of their hospitalization and received blood tests and the CGA. A total of 184 patients was included. The mean age was 84.6 years old and 61.6% were female. The analysis revealed arm circumference and level of frailty were significant predictors of home discharge. Specifically, 90.1% of patients had discharged home if their upper arm circumference was more than 18.3 centimeters, and 36.4% was discharged home if it was less than 18.3 centimeters. Among older adults with the arm circumference more than 18.3 centimeters, 57% of robust older adults and 91.7% of prefrail or frail older adults were discharged home. These robust older adults had relatively better daily living ability, and discharged to other facilities rather than home for rehabilitation. As many frail older adults are discharging to their own home, clinicians need to pay more attention to them. Also it is encouraged to perform the CGA, which includes nutritional assessment in it, for inpatient older adults.

